# Women’s experience of unintended pregnancy and changes in contraceptive methods: evidence from a nationally representative survey

**DOI:** 10.1186/s12978-022-01492-w

**Published:** 2022-09-01

**Authors:** Md Nuruzzaman Khan, M. Mofizul Islam

**Affiliations:** 1 Department of Population Science, Jatiya Kabi Kazi Nazrul Islam University, Mymensingh, 2222 Bangladesh; 2 Department of Public Health, La Trobe University, Melbourne, 3086 Australia

**Keywords:** Unintended pregnancy, Contraceptive, Mistimed pregnancy, Switching patterns of contraception, Bangladesh

## Abstract

**Background:**

Ineffective or no use of contraception following an unintended pregnancy contributes to a subsequent unintended pregnancy. This study aimed to determine whether women’s experiences of unintended pregnancies affect changing their contraceptive using patterns.

**Methods:**

We analysed the 2017/2018 Bangladesh Demographic and Health Survey data. The contraceptive switching pattern was computed by comparing women’s contraceptives using data before and after pregnancy. Women were categorised into the following three groups, depending on their patterns of contraceptive use before and after pregnancy: no change, if there were no change in contraceptive using pattern; switched to higher effective contraceptives, if changed from pre-pregnancy less effective contraceptives to post-pregnancy more effective contraceptives; switched to less effective contraceptives, if changed from pre-pregnancy more effective contraceptives to post-pregnancy less effective contraceptives. Women’s intention in the most recent pregnancy was our primary explanatory variable, classified as wanted, mistimed and unwanted. Multinomial multilevel logistics regression was used to determine the association between women’s intention in the most recent pregnancy and women’s contraceptive methods switching patterns from before to after pregnancy.

**Results:**

Around 20% of the most recent pregnancies that ended with a live birth were unintended at conception. No contraceptive use was reported by 37% of women before their pregnancies which decreased to 24% after pregnancies. Overall, around 54% of women who reported no contraceptive use before pregnancy used modern contraceptives after pregnancy. The rate was higher among women who experienced unwanted pregnancy (73.4%) than mistimed (58.8%) and wanted (53.4%) pregnancy. Experience of mistimed pregnancy was associated with a higher likelihood of no contraceptive change (aOR, 1.84, 95% CI 1.41–2.39) and switching to less effective contraceptives (aOR, 1.58, 95% CI 1.10–2.26) than switching to more effective contraceptives. However, unwanted pregnancy was not associated with any significant change in contraceptives use from before to after pregnancy.

**Conclusion:**

Experience of unintended pregnancy did not change women’s contraception using patterns, which indicates the risk of repeat unintended pregnancies and associated adverse consequences, including maternal and child morbidity and mortality. Policies to ensure access to and use of modern contraceptives among women facing unwanted or mistimed pregnancies are recommended.

## Plain Language summary

Unintended pregnancy is considered a public health challenge in low- and middle-income countries (LMICs). Around 89% of the global occurrences of unintended pregnancies occur in LMICs. In absolute numbers, this is equivalent to nearly 88 million pregnancies. Adverse pregnancy consequences, including bleeding, haemorrhage and maternal mortality are common in this group. The adverse consequences are even higher among women facing unintended pregnancies more than once, i.e., repeat unintended pregnancies, which represent around half of the total occurrences of unintended pregnancies in LMICs. Ensuring proper access to effective contraceptives following birth is key to reducing repeat unintended pregnancies and associated adverse consequences. It is critical to know the patterns of women’s pre-pregnancy contraceptive methods use and whether experiences of unintended pregnancies affect their contraceptive methods uptake and their types. However, this data is completely lacking in the context of LMICs. Using nationally representative survey data from Bangladesh, we examined women’s contraceptive switching patterns from before to after pregnancy and their association with pregnancy types. We found increased likelihood of no change in contraceptive using pattern or switching to less effective contraceptives among women after they experienced mistimed pregnancy compared to those whose pregnancy was wanted. This pattern increases the risk of repeat unintended pregnancies in Bangladesh and associated adverse consequences, including maternal and child morbidity and mortality.

## Introduction

The occurrences of unintended pregnancy are common in low- and middle-income countries (LMICs). It is estimated that around 88 million of the total 99 million unintended pregnancies occur in LMICs [1]. Although a precise estimate is lacking, a substantial proportion of these unintended pregnancies occur among women with prior experience of one or more unintended pregnancies [2]. Unintended pregnancy is associated with several adverse maternal and child health outcomes, including maternal and child mortality [2, 3]. Women who experience unintended pregnancy hold negative feelings about their pregnancies, have prior negative experiences with pregnancy, relatively low education status and socio-economic conditions [4]. As a result, they do not access the necessary maternal healthcare services, which is a principal reason for adverse consequences [5]. Negative feelings about pregnancy are even greater among women facing repeat unintended pregnancies. Moreover, repeat unintended pregnancy is also characterised by short birth intervals, relatively high number of births, undernutrition, and prior unintended pregnancy-related complications [6–8]. Consequently, adverse consequences are substantially high among women who experienced repeat unintended pregnancies.

Ensuring proper access to effective contraceptives following birth is key to reducing the repeat unintended pregnancy and associated adverse consequences [9]. However, this is still a challenge, particularly for socio-economically disadvantaged women as well as those living in rural areas, although successful implementation of the Millennium Development Goals helped increase contraceptive use substantially in LMICs (from 52 to 62% in 2015) [10]. Limited choices and access to contraceptives, fear or experience of side effects, cultural or religious opposition, poor quality of services, and gender-based barriers are precursors to non-use or traditional or less effective contraceptives use [11]. In fact, around half of married women of reproductive age living in LMICs do not have proper access to modern contraception (e.g., the pill, injectables, condoms, sterilization, intra-uterine devices, and implants) [10]. Other factors found to be associated with types of contraception use are future fertility preference and socio-demographic characteristics including women’s age, education level of women and their partners, socio-economic status, and mass media exposure [12–17].

To ensure effective contraceptives use among women with a history of unintended pregnancy, we need to know their pre-pregnancy contraceptive methods use and whether experiences of unintended pregnancies affect their contraceptive methods uptake and/or their types. However, as far as we know, these aspects have not been investigated in the context of LMICs, even though up to 43% of overall pregnancies are unintended [1], and the first occurrence of unintended pregnancy is a predictor of future unintended pregnancies [18, 19]. Although few studies reported an increased likelihood of modern contraceptive uptake among women with a history of unintended pregnancy, they did not compare with the pre-pregnancy contraceptive types [20–24]. Therefore, this study aimed to determine women’s contraceptive switching patterns from before to after pregnancy and if they differ among women who experienced unintended pregnancies.

## Methods

### Study overview

This study draws on data from publicly available nationally representative 2017/2018 Bangladesh Demographic and Health Survey (BDHS). It is a cross-sectional survey administered by the National Institute of Population Research and Training (NIPORT). Technical assistance to conduct this survey was provided by the ICF International of Calverton, Maryland, USA. The survey was based on a two-stage stratified sampling of households. At the first stage, 675 enumeration areas (clusters) were drawn from a list of 296,718, which was prepared for the national population and housing census conducted in 2011 by the Bangladesh Bureau of Statistics [25]. Of the 675 enumerations areas, 672 were finally selected and the remaining three were excluded because of a flood. At the second stage, 30 households from each enumeration area were selected by systematic random sampling, and all married women aged 15–49 years in the selected households were included. More information regarding the survey sampling procedure and collected data have been published in the BDHS survey report [26].

### Sample

**Fig. 1:**
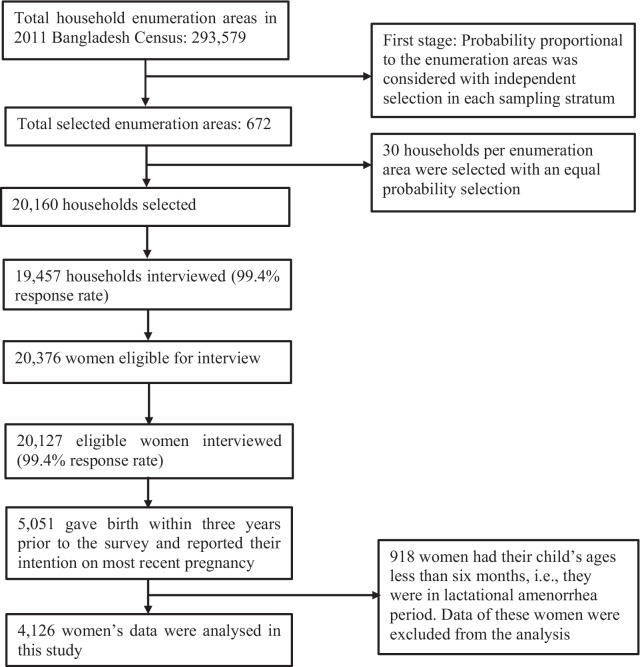
Sampling strategy of the 2017/2018 BDHS and sample selection procedure for this study

Of the 20,127 reproductive-aged married women interviewed in the 2017/2018 BDHS, data of 4126 women were included in the analysis (Fig. 1). The inclusion criteria were (i) fecund women (by birth) who were not pregnant at the time of the survey, (ii) had at least one birth in the 3 years preceding the survey (from whom the pregnancy intention data were collected), (iii) not in the infertile period due to absence of ovulation and (iv) who had at least one episode of sexual intercourse in the month preceding the survey. Women’s responses to the following three questions were used to determine the ovulation status and the month in which women became fertile again: (i) have you had menstrual bleeding?; (ii) are you giving regular supplementary foods or fruits or fluids to your baby in addition to breastfeeding?; and (iii) is your infant older than 6 months of age? [27] Women who answered negatively to each of these three questions were identified as infertile due to the absence of ovulation during the survey and excluded from the analysis [27].

### Outcome variable

The pattern of women’s contraceptive switch from before to after pregnancy is our outcome variable. The variable was generated by comparing women’s contraceptive using patterns at two different times: (i) at the month of the most recent pregnancy that ended with live birth (will be expressed as *before pregnancy* hereafter) and (ii) at the month when women became fertile again following the live birth of the most recent pregnancy (will be expressed as *after pregnancy* hereafter). The BDHS recorded these data using the calendar approach, which is a validated approach and used worldwide. In this approach, women’s monthly reproductive history, including contraceptive methods, pregnancy and birth from the survey dates to the previous 5 years was recorded [28]. Women were asked to report the names of their monthly contraceptive methods from a list that included: no method use, oral contraceptive pill, injections, condoms, female sterilization, male sterilization, intrauterine device, implant/Norplant, periodic abstinence and withdrawal. A free-text response option was also given to record if method(s) of contraceptives used were not on the list. Where women reported multiple contraceptive methods, their most frequently used method was selected. Using this data, we first determined women’s contraceptive methods using patterns before and after the most recent pregnancy that ended with live birth. We then classified them as no contraceptive use (if no methods were used), traditional contraceptives use (used periodic abstinence and/or withdrawal) and modern contraceptives use (used oral pills, injections, condoms, male and female sterilization, intra-uterine devices and/or implants). Contraceptive switching patterns were then determined by comparing types of contraceptives used before and after pregnancy. The variable was categorised as follows: (i) no change (if women reported the same contraceptive method use before and after pregnancy), (ii) switched to higher effective contraceptives (if women switched from pre-pregnancy less effective contraceptive methods to post-pregnancy more effective contraceptive methods), and (iii) switched to less effective contraceptives (if women switched from pre-pregnancy more effective contraceptive methods to post-pregnancy less effective contraceptive methods). International guidelines were followed to determine the effectiveness of contraceptive methods for this comparison [29].

### Exposure variables

The main exposure variable was the intention of the most recent pregnancy. This information was collected from women who had at least one live birth within 3 years prior to the survey. Women were asked: (i) when you became pregnant with (name of the last child born within 3 years of the survey date), did you want to get pregnant at this time? If women responded affirmatively, responses were categorised as wanted pregnancy. If women responded negatively, their pregnancies were identified as unintended. They were then asked: did you want to have a baby later on, or did you not want any more children? Responses were recorded as “later” and “no more/none”. Responses were then categorised as mistimed pregnancy (if the answer was “later”) and unwanted pregnancy (if the answer was “no more/none”).

Potential confounding variables were identified by a comprehensive literature search on the determinant factors of contraception use [12–17]. Respondents’ level factors included were women’s age at birth of the last child (≤ 19, 20–34, ≥ 35), desire for more children (within 2 years, after 2 years, and no more children wanted), women’s educational status (no formal education, primary, secondary, and higher), and parity (one child, two children, three or more children). Husbands’ educational status (no formal education, primary, secondary, and higher), occupation (agricultural worker, services and non-agricultural labor, business, and others), and household wealth quintile (poorest, poorer, middle, richer, and richest) were also identified as potential covariates. Other factors were residential locations (urban, rural) and seven administrative divisions of residence (Barishal, Chattogram, Dhaka, Khulna, Mymensingh, Rajshahi, Sylhet).

### Statistical analysis

We used descriptive statistics to characterise the demographic profile of women and two-sample proportion tests to assess the significance of the differences in contraceptive methods uses before and after the most recent pregnancy. In the BDHS, respondents included are nested in the households, and households are nested in the clusters. Therefore, responses from the same household and cluster are more likely to be similar and behave more alike than a different household and cluster. Multilevel regression is an appropriate approach for this type of hierarchical data [30]. Therefore, we used multinomial multilevel logistic regression to examine the association between the intention of the most recent pregnancy occurring within the 3 years prior to the survey and women’s contraceptive methods switching patterns from before to after pregnancy. The presence of multicollinearity was checked among variables using Variance Inflation Factors (VIF) at a cut-off point of 10. If VIF was more than 10, the relevant variable was deleted. Results were reported as adjusted odds ratios (aOR) with 95% confidence intervals (95% CI). All statistical analyses were conducted using Stata software version 15.1 (Stata Corp, College Station, Texas, USA).

## Results

Over one-fifth of 4126 women included in the analyses reported their last pregnancies were unintended (Table 1). At the time of the survey, around 70% were in the 20–34 age group, and almost half had completed secondary education. The mean number of children per woman was 2.11, and over 61% of women reported having at least two children. Around half of women reported that they did not want to have any child in the future. Nearly 75% of women were residing in rural areas.

**Table 1:** Characteristics of women who gave at least one live birth within 3 years prior to the 2017/2018 BDHS survey and were not in the post-partum amenorrhea period at the time of survey (n = 4126)

Variables	Percent (95% CI)
Pregnancy intention at conception	
Wanted	79.2 (77.9–80.5)
Mistimed	13.2 (12.2–14.4)
Unwanted	7.6 (6.7–8.5)
Women’s age (in years) at birth of last child	
Mean (± SE)	**23.92 (± 0.09)**
≤ 19	25.9 (24.4–27.5)
20–34	69.9 (68.2–71.5)
≥ 35	4.2 (3.6–4.9)
Women’s education	
Mean years of schooling (± SE)	**6.98 (± 0.09)**
No formal education	6.3 (5.4–7.3)
Primary	27.4 (25.5–29.3)
Secondary	49.2 (47.3–51.2)
Higher	17.1 (15.5–18.8)
Maternal parity	
Mean number (± SE)	**2.11 (± 0.02)**
1 child	38.9 (37.3–40.6)
2 children	32.8 (31.2–34.4)
3 and more children	28.3 (26.6–30.1)
Pregnancy desire	
Wants within 2 years	4.8 (4.1–5.7)
Wants after 2 years	46.0 (44.2–47.8)
Wants no more	49.2 (47.4–51.0)
Husbands’ education	
Mean years of schooling (± SE	**6.76 (± 0.11)**
No formal education	14.1 (12.6–15.8)
Primary	33.9 (32.0–35.9)
Secondary	33.7 (31.9–35.5)
Higher	18.3 (16.7–20.0)
Husbands’ occupation	
Agricultural work	19.3 (17.5–21.2)
Physical labourer	53.3 (51.1–55.4)
Services	5.8 (5.0–6.8)
Business	21.0 (19.4–22.7)
Other	0.6 (0.4–0.9)
Household wealth quintile	
Poorest	20.6 (18.5–22.8)
Poorer	20.7 (19.1–22.4)
Middle	18.7 (17.1–20.4)
Richer	20.2 (18.4–22.1)
Richest	19.9 (17.9–22.1)
Place of residence	
Urban	26.9 (25.1–28.8)
Rural	73.1 (71.2–74.9)
Division of residence	
Barishal	5.6 (5.0–6.2)
Chattogram	20.8 (19.0–22.8)
Dhaka	25.9 (24.0–27.8)
Khulna	9.1 (8.2–10.2)
Mymensingh	8.5 (7.6–9.5)
Rajshahi	11.9 (10.6–13.4)
Rangpur	10.7 (9.6–11.9)
Sylhet	7.5 (6.6–8.5)

n = total number of women included in the study. All percentages are weighted

Women’s before and after pregnancy contraceptive using patterns are presented in Table 2. Pills were the main contraceptives both before and after the pregnancy, and around 40% of women used them. There had been no significant changes in the specific contraceptives used before and after the pregnancy, except the no contraceptive use and the Norplant. Women who used *no contraceptives* declined from 37.2% before pregnancy to 24.4% after pregnancy, and women who used Norplant increased from 0.6 to 2.1%. Comparison of three classes of contraceptives in terms of their uses before and after pregnancy showed a significant difference in modern contraceptives and no contraceptive use groups (Table 2).

**Table 2:** Distribution of contraceptive methods used before and after the most recent pregnancy that ended with live birth, Bangladesh Demographic and Health Survey, 2017/2018, (n = 4126)

Contraceptives	Before pregnancy (%)	After pregnancy (%)	*p*
No contraceptive use	1536 (37.2)	1004 (24.4)	0.01
Pill	1622 (39.3)	1576 (38.4)	0.56
Intra-uterine device	3 (0.07)	19 (0.5)	0.16
Injectable	388 (9.4)	522 (12.6)	0.15
Condom	347 (8.4)	500 (12.1)	0.09
Female sterilization	0 (0.0)	98 (2.4)	–
Male sterilization	0 (0.0)	10 (0.2)	–
Periodic abstinence/Rhythm	140 (3.8)	189 (4.6)	0.72
Withdrawal	64 (1.6)	120 (2.9)	0.59
Norplant	26 (0.6)	88 (2.1)	0.01
Across three groups of contraceptive methods^++^			
No contraceptive methods	1536 (37.2)	1004 (24.3)	0.01
Traditional contraceptive methods	204 (5.0)	309 (7.5)	0.26
Modern contraceptive methods	2386 (57.8)	2813 (68.2)	0.01

^**++**^Classification is based on guidelines of the World Health Organization and the Centre for Diseases Control. All estimates are weighted

**Fig. 2:**
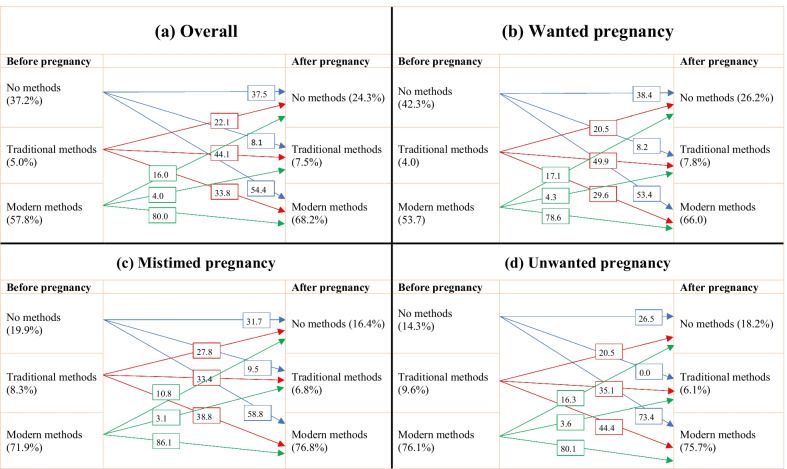
Contraceptive methods use before and after pregnancy across types of pregnancy intentions

Women’s contraceptive methods switching patterns across three types of intentions of the most recent pregnancies are presented in Fig. 2. Around 73% of women who experienced unwanted pregnancy and 59% of women who experienced mistimed pregnancy switched from no contraceptive to modern contraceptive methods.

Table 3 shows the unadjusted associations between the contraceptive methods switching patterns and women’s intention to most recent pregnancy at conception. Likelihoods of no change of contraceptive methods and switching to less effective contraceptive methods were significantly higher among respondents who reported their most recent pregnancy as mistimed and unwanted at conception than women with wanted pregnancy at conception.

**Table 3:** Multilevel multinomial logistic regression model with pregnancy intention as the sole correlate of the change in contraceptive methods use, from before to after the most recent pregnancy

	Changes in the contraceptive methods before to after pregnancy (reference: switched to a higher effective method)			
	No change in methods		Switched to a less effective methods	
	Odds ratio (95% CI)	*p*	Odds ratio (95% CI)	*p*
Most recent pregnancy intention at conception: wanted conception (ref)	1.00	–	1.00	–
Mistimed conception	2.09 (1.65–2.65)	< 0.01	1.92 (1.38–2.68)	< 0.01
Unwanted conception	2.12 (1.54–2.91)	< 0.01	2.71 (1.80–4.06)	< 0.01
Constant	2.17 (1.99–2.36)	< 0.01	0.44 (0.39–0.50)	< 0.01
Random effects^a^				
Cluster-level variance (SE)^b^	0.08 (0.05)***			
Log-likelihood for fixed effects to random effects model	1423.29***			
Log-likelihood ratio test for the null model to the random effects model (Chi-square)^c^	62.97***			

^a^We assume that the within cluster-level random effects are equal for the ‘no change of contraceptive method’ and ‘switch to a less effective contraceptive methods’; therefore, only between cluster-level variance estimates are reported

^b^Significance of random effects evaluated by comparing the model with a similar one in which random effects were constrained to zero

^c^Compared to the null model with no-covariates. ***p < *0.01*

These associations persisted for mistimed pregnancy following multivariable adjustment (Table 4), while the associations for unwanted pregnancy disappeared. In the adjusted model, compared to wanted pregnancy, a mistimed pregnancy had 84% (aOR, 1.84; 95% CI 1.41–2.39) and 58% (aOR, 1.58; 95% CI 1.10–2.26) higher likelihoods of no change in contraceptive methods and switching to a less effective contraceptive methods, respectively, than switching to higher effective contraceptive methods. In the regression model run with the variable, unintended pregnancy (combining mistimed and unwanted pregnancies), aOR was 1.69 (95% CI 1.35–2.13) for no change in contraceptive methods and 1.57 (95% CI 1.16–2.12) for switching to less effective contraceptive methods than switching to higher effective contraceptive methods (results not shown in table).

**Table 4:** Odds ratios from multilevel multinomial logistic regression examining the association between switching patterns of contraceptive methods and women’s pregnancy intention at conception

	Changes in the contraceptive methods before to after pregnancy (Reference: switched to higher effective methods)			
	No change in methods		Switched to less effective methods	
	Odd ratio (95% CI)	*p*	Odd ratio (95% CI)	*p*
Most recent pregnancy intention at conception: wanted conception (ref)				
Mistimed conception	1.84 (1.41–2.39)	< 0.01	1.58 (1.10–2.26)	< 0.01
Unwanted conception	1.36 (0.91–2.04)	0.13	1.45 (0.88–2.38)	0.14
Desire for pregnancy in the future: wants within 2 years (ref)				
Wants after 2 years	0.98 (0.68–1.40)	0.90	1.18 (0.64–2.17)	0.60
Wants no more	1.10 (0.74–1.64)	0.64	1.40 (0.76–2.60)	0.28
Women’s age at birth of their last child: ≤ 19 years (ref)				
20–34 years	1.24 (1.01–1.52)	< 0.05	1.51 (1.08–2.11)	< 0.05
≥ 35 years	1.37 (0.80–2.34)	0.24	1.21 (0.59–2.48)	0.60
Parity: 1 child (ref)				
2 children	3.41 (2.66–4.37)	< 0.01	4.91 (3.40–7.10)	< 0.01
3 children	3.03 (2.21–4.16)	< 0.01	5.44 (3.52–8.40)	< 0.01
Women’s education: no formal education (ref)				
Primary	1.15 (0.76–1.74)	0.50	1.25 (0.74–2.12)	0.40
Secondary	1.25 (0.81–1.94)	0.31	1.43 (0.83–2.49)	0.20
Higher	1.34 (0.81–2.20)	0.26	1.66 (0.85–3.25)	0.14
Husbands’ education: no formal education (ref)				
Primary	1.02 (0.78–1.33)	0.88	0.95 (0.67–1.34)	0.79
Secondary	0.96 (0.71–1.28)	0.78	1.11 (0.75–1.63)	0.09
Higher	0.95 (0.65–1.38)	0.78	0.96 (0.57–1.61)	< 0.01
Husbands’ occupation: agricultural worker (ref)				
Labourer	1.17 (0.93–1.45)	0.18	1.29 (0.94–1.78)	0.11
Services	1.21 (0.79–1.86)	0.37	0.85 (0.44–1.66)	0.64
Business	0.96 (0.73–1.26)	0.79	1.08 (0.74–1.57)	0.70
Other	0.73 (0.25–2.08)	0.55	0.74 (0.16–3.53)	0.71
Wealth status: poorest (ref)				
Poorer	1.15 (0.89–1.49)	0.29	1.24 (0.88–1.75)	0.23
Middle	1.20 (0.91–1.58)	0.20	1.14 (0.78–1.66)	0.51
Richer	1.18 (0.88–1.57)	0.26	1.19 (0.80–1.79)	0.39
Richest	1.41 (1.00–1.99)	< 0.05	0.99 (0.61–1.61)	0.96
Place of residence: urban (ref)				
Rural	1.11 (0.91–1.35)	0.32	1.31 (0.97–1.77)	0.08
Division of residence: Barishal (ref)				
Chottogram	0.58 (0.41–0.81)	< 0.01	0.62 (0.39–0.96)	< 0.05
Dhaka	1.05 (0.71–1.56)	0.80	0.84 (0.51–1.38)	0.50
Khulna	0.79 (0.54–1.16)	0.23	1.17 (0.69–2.00)	0.56
Mymensingh	0.60 (0.43–0.85)	< 0.01	0.66 (0.41–1.05)	0.08
Rajshahi	0.63 (0.43–0.91)	< 0.05	0.56 (0.34–0.95)	< 0.05
Rangpur	0.54 (0.37–0.78)	< 0.01	0.60 (0.36–0.99)	< 0.05
Sylhet	0.53 (0.37–0.78)	< 0.01	0.44 (0.27–0.71)	< 0.01
Random effects^a^				
Cluster-level variance (SE)^b^	0.03 (0.04)***			
Log-likelihood for fixed effects to random effects model	609.44***			
Log-likelihood ratio test for the null model to random effects model (Chi-square)^c^	1624.59***			

^a^We assume that the within cluster-level random effects are equal for the ‘no change of contraceptive method’ and ‘switched to a less effective contraceptive methods’; therefore, only between cluster-level variance estimates are reported

^b^Significance of random effects evaluated by comparing the model with a similar one in which random effects were constrained to zero

^c^Compared to the null model with no-covariates

Of the covariates, women of 20–34 years age-group and increasing parity had higher likelihoods of no change in contraceptive methods and switching to a less effective contraceptive methods. Lower likelihoods of no change in contraceptive methods or switching to less effective contraceptive methods were found among women in five of the seven divisions, namely Chattogram, Mymensingh, Rajshahi, Rangpur and Sylhet division than women in the Barishal division.

## Discussion

This study explored changes in women’s contraceptive methods uptake before and after pregnancy and determined their association with their most recent pregnancy types. To our knowledge, this study is the first of its kind in the context of a LMIC. The findings indicate that unintended pregnancy was relatively high in Bangladesh. After experiencing unintended pregnancies, women were more likely to bring no change in contraceptives or switch to relatively low effective contraceptives than switching to relatively high effective contraceptives. This pattern of contraceptive use is likely to cause further unintended pregnancies and associated adverse outcomes, including maternal and child morbidity and mortality [31, 32].

This study found around 20% of the most recent pregnancies that ended with live births among married women of reproductive age in Bangladesh were unintended at conception, which is approximately 5% less than the previously available estimate based on the 2014 BDHS [22]. Around 37% of women in our study did not use contraceptives; this is consistent with the average rate of no contraception use in Bangladesh [26]. This rate decreased to 24% after pregnancy. However, our multivariable analysis suggests increased likelihoods of no change in contraceptive methods and switching to less effective contraceptive methods following the occurrence of a mistimed pregnancy but insignificant changes in likelihood among women who experienced an unwanted pregnancy. The lack of relevant literature limits our ability to compare these findings.

Mistimed pregnancies in Bangladesh and other LMICs are most often characterised by lower maternal age [22, 33]. These pregnancies increase women’s psychological stresses in the perinatal [34] and postnatal periods [35] and restrict them from accessing maternal healthcare services that should be a good source of knowledge to switch to modern and effective contraceptives following the end of their current pregnancy [36]. In the context of unwanted pregnancies, these difficulties are added to their previous experiences of pregnancies as these often occur among women higher in age and parity [22]. Together these work as barriers for women to access maternal healthcare services [3]. A relatively low interaction with healthcare services together with depression in the postnatal period could reduce the likelihood of visiting healthcare centers in the post-partum period. As a result, women who experienced unintended pregnancies do not change their contraceptive behaviors or switch to a less effective contraceptive method.

The current approach to family planning and maternal healthcare services during post-partum visits and contraceptive methods uptake may act as a deterrent. In Bangladesh, there is inadequate coordination in the way maternal healthcare and family planning services are delivered. Visits to healthcare facilities during and early pregnancy are often treated as pregnancy-related, and only the post-partum visits at the fourth week of live birth are dedicated to providing family planning services [37]. Shockingly, the prevalence of 4th-week visits is only 0.2% in Bangladesh and the likelihood of this visit is around 37% lower among women facing an unintended pregnancy than the women having wanted pregnancy [38]. Moreover, health and family planning services are delivered disjointly [37]. Although integration of these services was emphasized in recent 5-year plans [39, 40], this has been vaguely implemented at the field level. This disjoint service delivery approach puts the responsibility of much of the contraception services with the family planning workers. As a result, service modalities for contraception services have not been properly developed in the sub-districts and lower-level health centers. For instance, in many locations, there is no separate corner to discuss family planning-related issues privately though this is a culturally sensitive topic in Bangladesh [22]. Maternal healthcare service providers pay less interest in providing family planning services. Also, the healthcare facilities in Bangladesh usually follow a visits-based approach to provide maternal healthcare services (women received care only if they visit healthcare centers), and there exists no help for couples who are unable to attend healthcare facilities [38]. In addition, there is a lack of provision in Bangladesh to record women’s pregnancy intention at the earlier stages of pregnancy [22]. Consequently, maternal healthcare providers do not know about the women who are unlikely to access maternal healthcare and family planning services. These challenges add up to the commonly reported challenges to access to and use of contraceptives in Bangladesh, including infrequent family planning visits and substandard quality of services [37]. Together, these indicate a massive barrier to change women’s contraceptive behaviour following their unintended pregnancies [24, 38, 41, 42].

Women of 20–34 years old or those who had two or more children were significantly more likely to make no change in contraceptive methods or switch to less effective contraceptive methods. Although further examination as to why these variables behave across women’s pregnancy intentions is beyond the scope of this study, some possible explanations are that these women want to have another child shortly after their current pregnancy [43], and/or negative effect of current unintended pregnancy including depression and anxiety [22]. Also, findings regarding the association between high parity, which is an important determinant of unintended pregnancy, and no change/switch to a less effective contraceptive method may indicate these women are indifferent about their fertility and number of births [43, 44]. This indifference may be connected to their relatively low awareness, autonomy as well as situational factors, including rurality, which affects the availability of and accessibility to healthcare services [43].

To our knowledge, this is the first study in the context of an LMIC that used data from the unique representative survey and examined potential countervailing impacts of women’s pregnancy intention on contraceptive methods switching patterns from before to after pregnancy. A relatively large sample size helped us to assess the actual effect and therefore draw useful conclusions. Moreover, appropriate statistical adjustments for the survey design and the confounding effects make the findings reliable. This study also has several limitations. As this is a cross-sectional study, the relationship reported is correlational only and not causal. Also, the data were based on participants’ self-report with no scope for validation by the interviewers and may be subject to reporting errors. Moreover, pregnancy intention data were collected retrospectively for births occurring up to 3 years prior to the survey, which is also subject to recall bias. However, this bias is likely to be random. Data were collected only from women who had given birth and did not include the pregnancies that were terminated, resulting in underreporting of the actual occurrence of unintended pregnancies. Moreover, after the most recent pregnancy, contraceptive use was determined for the most recent month following the end of the post-partum amenorrhea period. There is a possibility that some women would uptake more effective contraceptive methods in the subsequent months. But as this data was not available, we were unable to account for that in the analysis. Additionally, some confounding factors considered in the analysis could be different at the time when women experienced unintended pregnancy and at the time when they returned in the fertile period following the end of the previous pregnancy. However, it was not possible to identify these variations from the survey data we analysed. Furthermore, some other factors such as cultural and behavioural characteristics, previous unintended pregnancies and religious convictions may affect contraceptive methods-changing behaviours, which we could not account for, as no data on these are available in the BDHS survey.

## Conclusion

This study provided quantitative evidence that there was a lower likelihood among women whose most recent pregnancy was unintended to change their pre-pregnancy contraceptive methods or switch to less effective contraceptive methods. Such practices placed these women at an increased risk of future unintended pregnancies. Increased provision of counselling to women who have had an unintended pregnancy is required to change their contraceptive methods using behaviour. Also, the coverage of contraception services, particularly the long-acting reversible contraception (LARC) methods, needs to be increased. Integrated family planning services with mainstream maternal healthcare services and earlier detection of pregnancy are recommended.

## Data Availability

The data that support the findings of this study are available from the DHS Program, but restrictions apply to the availability of these data, which were used under license for the current study, and so are not publicly available. Data are, however, available from the authors upon reasonable request and with permission of the DHS Program.

## Abbreviations

LLMICs: Low- and middle-income countries
BDHS: Bangladesh Demographic and Health Survey
NIPORT: National Institute of Population Research and Training
OR: Odds ratios
AIC: Akaike Information Criteria
BIC: Bayesian Information Criteria
